# Supplementary material for: Correlation between Histopathological Prognostic Tumor Characteristics and [18F]FDG Uptake in Corresponding Metastases in Newly Diagnosed Metastatic Breast Cancer
Source: Diagnostics (Basel). 2024 Feb 14;14(4):416. doi: 10.3390/diagnostics14040416 (PMC10887896; doi:10.3390/diagnostics14040416)

Extra information on Supplemental Data

Supplemental S1. [^{18}F]FDG Uptake and SUV Measures

We assessed the effect of SUV calculation method (SUV_{max}, SUV_{peak}, and SUV_{mean}) on per lesion [^{18}F]FDG uptake in all 188 patients. There was a high correlation between SUV_{max} and SUV_{peak} (Pearson's r=0.98; p<0.001), SUV_{max} and SUV_{mean} (Pearson's r=0.93; p<0.001), and also between SUV_{peak} and SUV_{mean} (Pearson's r=0.94; p<0.001; Supplemental Figure S1).

Supplemental Figure S1. Correlation of A) [^{18}F]FDG uptake (SUV_{max}) and [^{18}F]FDG uptake (SUV_{peak}), B) [^{18}F]FDG uptake (SUV_{max}) and [^{18}F]FDG uptake (SUV_{mean}), and C) [^{18}F]FDG uptake (SUV_{peak}) and [^{18}F]FDG uptake (SUV_{mean}).

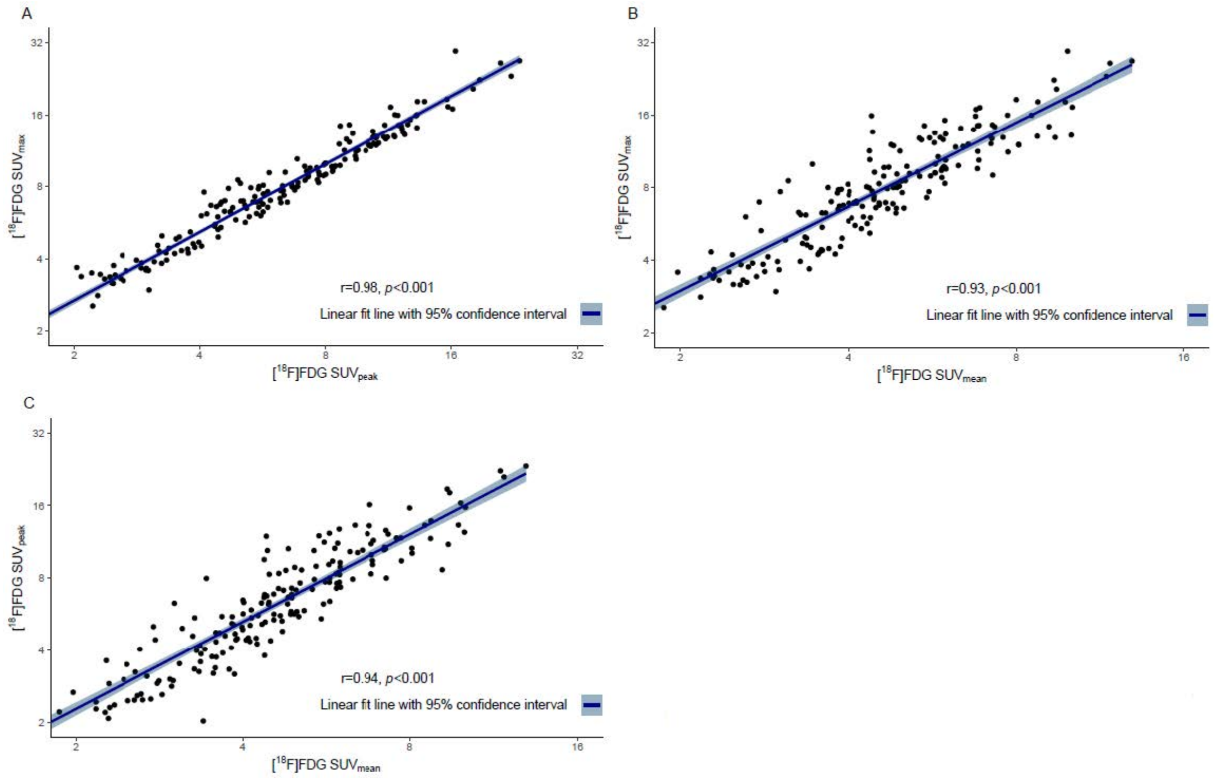

Supplement: Supplementary file 1 [file diagnostics-14-00416-s001.zip › diagnostics-2806305-supplemental_1.pdf]
