# Supplementary material for: Correlation between Histopathological Prognostic Tumor Characteristics and [18F]FDG Uptake in Corresponding Metastases in Newly Diagnosed Metastatic Breast Cancer
Source: Diagnostics (Basel). 2024 Feb 14;14(4):416. doi: 10.3390/diagnostics14040416 (PMC10887896; doi:10.3390/diagnostics14040416)

Supplemental S2. Correlation of [18F]FDG uptake (SUVmax) uptake and tumor lesions on diagnostic CT (expressed as volume)

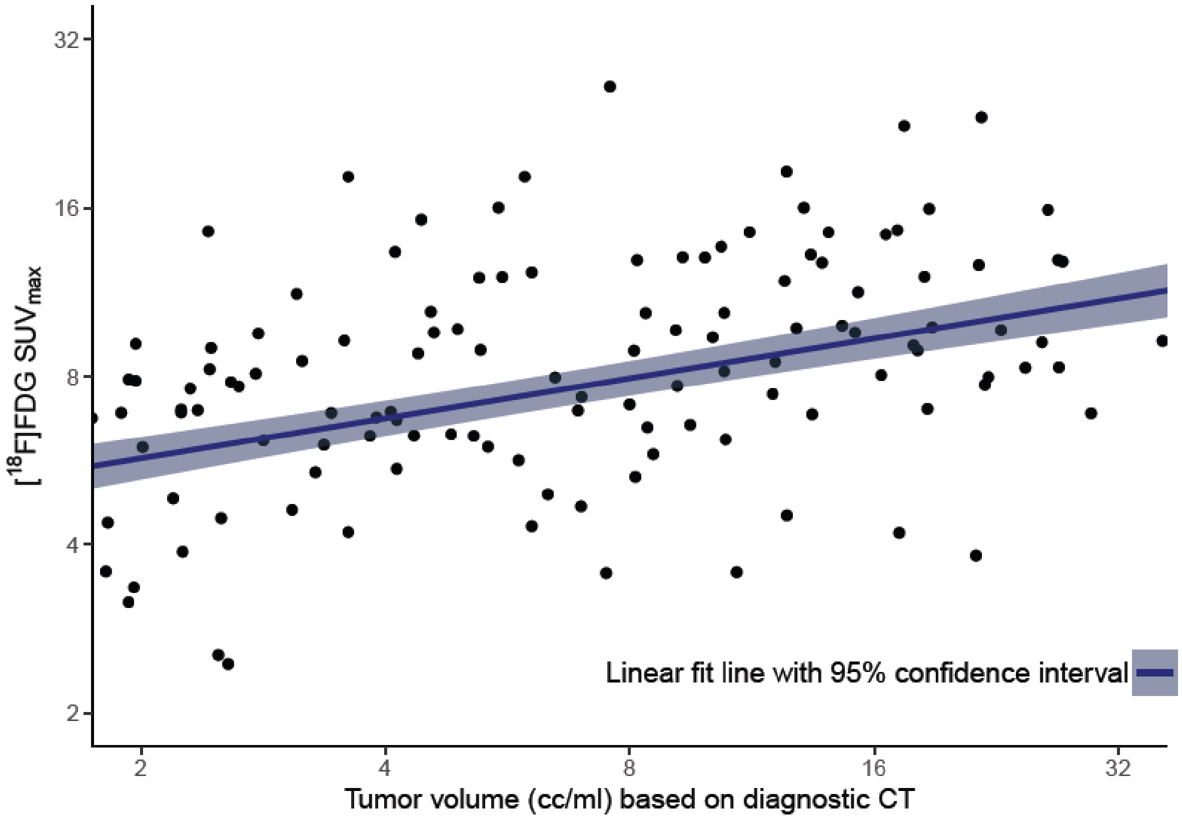

Supplement: Supplementary file 1 [file diagnostics-14-00416-s001.zip › diagnostics-2806305-supplemental_2.pdf]
