# Supplementary material for: Correlation between Histopathological Prognostic Tumor Characteristics and [18F]FDG Uptake in Corresponding Metastases in Newly Diagnosed Metastatic Breast Cancer
Source: Diagnostics (Basel). 2024 Feb 14;14(4):416. doi: 10.3390/diagnostics14040416 (PMC10887896; doi:10.3390/diagnostics14040416)
Supplement: Supplementary file 1 [file diagnostics-14-00416-s001.zip › diagnostics-2806305-Supplemental_3.pdf]

Supplemental S3. Effect Endocrine Treatment Before [^{18}F]FDG-PET

Fourteen of the 188 patients were treated with aromatase inhibitors and one patient with anti-estrogen as adjuvant therapy ≤7 days before [^{18}F]FDG-PET scan. None of the 188 patients used chemotherapy or targeted therapy during this period. Receiving aromatase inhibitors before [^{18}F]FDG-PET acquisition, may have possibly influenced tracer uptake (1). Therefore, we assessed the influence of adjuvant endocrine treatment on [^{18}F]FDG uptake. The use of adjuvant endocrine treatment shortly before [^{18}F]FDG-PET (n=15) did not affect [^{18}F]FDG uptake compared to [^{18}F]FDG uptake in patients without adjuvant treatment (n=173; $SUV_{max\ geom.\ mean}$ of 7.0 versus 7.5; p=0.707).

(1) Kurland BF, Gadi VK, Specht JM, Allison KH, Livingston RB, Rodler ET, et al. Feasibility study of FDG PET as an indicator of early response to aromatase inhibitors and trastuzumab in a heterogeneous group of breast cancer patients. EJNMMI Res. 2012;2:1.
