# Supplementary material for: Correlation between Histopathological Prognostic Tumor Characteristics and [18F]FDG Uptake in Corresponding Metastases in Newly Diagnosed Metastatic Breast Cancer
Source: Diagnostics (Basel). 2024 Feb 14;14(4):416. doi: 10.3390/diagnostics14040416 (PMC10887896; doi:10.3390/diagnostics14040416)
Supplement: Supplementary file 1 [file diagnostics-14-00416-s001.zip › diagnostics-2806305-supplemental_4.pdf]

Supplemental S4. Lesions not visible on [^{18}F]FDG-PET. Lesions without [^{18}F]FDG uptake above background on the PET scan. In 175/182 patients the lesion showed [^{18}F]FDG uptake above background on the PET scan. The remaining seven lesions were not visible on [^{18}F]FDG-PET

**Histological subtype**	**ER status**	**% ER**	**HER2 status**	**Location**	**Size on CT**
Invasive carcinoma NST	Positive	100	Negative	Axillary lymph node	<10mm
Invasive carcinoma NST	Positive	100	Negative	Ilium (bone)	not measurable
Invasive carcinoma NST	Positive	100	Negative	Liver	17mm
Invasive carcinoma NST	Positive	100	Negative	Liver	12mm
Invasive carcinoma NST	Positive	100	Negative	Breast skin	not measurable
Invasive carcinoma NST	Positive	100	Negative	Lung	14mm
Invasive lobular carcinoma	Positive	100	Negative	Abdominal skin	<10mm
